# Supplementary material for: Knowledge and attitudes of antenatal mothers towards intimate partner violence in the Gambia: A cross-sectional study
Source: PLOS Glob Public Health. 2024 Feb 15;4(2):e0001257. doi: 10.1371/journal.pgph.0001257 (PMC10868735; doi:10.1371/journal.pgph.0001257)
Supplement: Attachment — Submitted filename: Dear Editor.docx [file pgph.0001257.s002.docx]

Dear Editor,

**Rebuttal letter**

Thank you for considering our manuscript, " **Knowledge and attitudes of antenatal mothers towards intimate partner violence in The Gambia: a cross-sectional study in rural health facilities.**," for publication in Plos Global Public Health. We appreciate the feedback provided by the reviewers and are happy to address their concerns.

Reviewer 2 raised some interesting observations most of whom we agree with.

Below are some of the comments made line by line and the accompanying responses:

**Comment Line 49:** Could “issue” be missing from human rights?

Response: No “issue” is not missing, it is meant to ‘….. central human right and public health **Concern**

**Comment Line 75:** Please spell out ANC

**Response :** Done

Line 88: Sentence is a little unclear

Response: Rewritten

Line 93: Do you know what percentage of antenatal women utilize the public health facilities in URR? Do their demographics differ from the general antenatal population

Response: the demographic of antenatal attendees in this study does not differ from the general antenatal population

Line 95: “Purposively selected”, what was the reasoning behind this choice?

**Response:**

Line 110 – I don’t quite understand this sentence, did you ask 400 women to participate with 373 agreeing to participate

**Response:** Sentence is rewritten in simpler terms for easier comprehension

Line 128 – Who offered the pieces of paper? The research assistants or health care personnel?

**Response:** by the research assistants and it is now specified in the paper
Line 132 – I think it would be helpful to show some sample questions and possible answers, i.e. are these yes/no answers? Any missing, and if yes how was that handled?

**Response**: Sample questions and their responses can be seen in the results section, for example table 2. An no there were no missing responses as it was interviewer administered

Line 149 - Please spell out CRR.

**Response:** Done

Line 198 - I think there is an extra and “psychological and intimate partner violence”.

**Response:** Noticed and the extra is deleted

Line 203 – I don’t believe that this dichotomy of good vs. poor knowledge was introduced in the methods section.

Response: No, it wasn’t, now introduced in line 173

Line 218 – I don’t believe that the Likert scale is mentioned in the methods section. Also missing in the methods section is how positive and negative attitudes are defined.

**Response:** No, it wasn’t, now introduced in line 176

Line 224 – More detail on this table in the methods section would be appreciated.

**Response:** The details on this table are in the immediate text preceding it

Line 234 – I don’t believe the information presented in Figure 2 was introduced in the methods section.

**Response:** Now introduced in line 145

Line 238 – Please describe how many women raised these other suggestions.

**Response:**  Now described in line 258

Line 260 – Is the Nigerian study comparable to this study?

**Response:** We believe it is comparable to Nigeria as similar methodologies were employed, such as the data collection tool which were both adapted from WHO multicounty questionnaire on domestic violence. Plus, both countries have a similar literacy rate, the literacy rate in the Gambia is 64% whilst that of Nigeria is 61%. Religious similarities between northern Nigeria and the Gambia among other characteristics makes it comparable in our opinion.

Line 267 – This sentence is a little unclear.

**Response:** Clarified in line 286

Line 269 – Are you referring to the prevalence or knowledge levels in the WHO study?

**Response:** No, we are referring to this study in a new sentence in line 288

Line 282 – Missing reference.

Response: The statistic provided is quoted from this study and not citing a different study

Line 286 – Please describe this Nigerian study. Are these groups comparable?

**Response:** This line is not talking about any Nigerian study, it reads, “ To conclude, antenatal mothers’ knowledge on intimate partner violence must be raised in general regarding the different forms of violence to drive good attitudes towards IPV.”

Line 297 – This sentence is a little unclear.

**Response:** This sentence has been deleted from the article

Line 302 – Where does this statistic come from?

**Response:** The concerned studies are duly cited

Line 303 – Missing reference.

**Response: Citation inserted**

Line 304 – Did you mean undone rather than outdone?

**Response:** the sentence has been deleted in line 331

Line 308 – Missing reference

**Response: this sentence is talking about this study**

We hope that these responses address the concerns raised by the reviewers and demonstrate the value of our study for Plos Global Public Health. We appreciate the opportunity to revise and resubmit our manuscript for further consideration.

Sincerely,

Joseph W. Jatta
